# An above-knee compression garment does not improve passive knee joint position sense in healthy adults

**DOI:** 10.1371/journal.pone.0203288

**Published:** 2018-09-04

**Authors:** János Négyesi, Ali Mobark, Li Yin Zhang, Tibor Hortobagyi, Ryoichi Nagatomi

**Affiliations:** 1 Department of Medicine and Science in Sports and Exercise, Tohoku University Graduate School of Medicine, Sendai, Japan; 2 Division of Biomedical Engineering for Health & Welfare, Tohoku University Graduate School of Biomedical Engineering, Sendai, Japan; 3 Department of Sports Health Science, Faculty of Physical Education, Tanta University, Tanta, Egypt; 4 Center for Human Movement Sciences, University Medical Center Groningen, University of Groningen, Groningen, the Netherlands; University of Innsbruck, AUSTRIA

## Abstract

We determined the effects of wearing an above-knee compression garment (CG) on knee joint position sense. Healthy young adults (n = 24, age = 27.46 ± 4.65 years) performed a passive knee position-matching task on an isokinetic dynamometer with each leg separately. We determined the magnitude of compression by measuring anatomical thigh cross sectional area (CSA) in standing using magnetic resonance imaging. Wearing the CG compressed CSA by 2% (t = 2.91, p = 0.010, Cohen’s d = 0.68). Repeated measures ANOVA (rANOVA) with three repetition factors (condition: CG, no CG; leg: right dominant, left non-dominant; and target angles: 30°, 45°, 60°) revealed an effect of angles (p < 0.001), where the matching of knee joint position was more accurate at 60° compared to 30° and 45° (p < 0.001). However, CG did not reduce passive joint position sense errors. In fact, joint position error was less without CG (p = 0.014). In conclusion, while CG does compress the thigh it does not afford the purported benefits for proprioception as measured by a target-matching task in the present study.

## Introduction

Proprioception is a sense of position and motion of limbs and contributes to joint stability [1–4]. Braces, limb sleeves, and compression garments (CGs) increase joint stability and are also believed to enhance joint position sense [5, 6]. These prophylactic devices have become popular among athletes to improve athletic performance, reduce risks for injuries, and facilitate recovery from injuries [7]. It is speculated that CGs improve the sense of limb in space by stretching the skin which in turn augments the sense of movement [8], proprioceptive acuity [6], and by relieving muscle fatigue [6, 9]. However, the favourable effects of soft tissue compression are not consistent because limb compression and ischemia, phenomena also produced by CGs, reduced the discharge rate of Ia afferents and impaired joint position sense [10].

Knee joint proprioception is the perceived sense of knee joint position and movement in the joint [11]. Paralleling the inconsistencies of the physiological mechanisms of limb compression, the results are also contradictory concerning the effects of compression on knee joint position sense in individuals with [12, 13] and without an anterior cruciate ligament injury [14–16]. While some authors contend that the benefits of using CGs are related to the magnitude and uniformity of compression in the muscle produced by a CG [17, 18], others suggest the effectiveness of CGs and pressure are unrelated [7]. Another source of the inconsistencies could be related to mixing data from dominant versus non-dominant limbs in the analyses, as proprioceptive acuity is greater when target-matching is done with the non-dominant compared with the dominant limb [19–21].

Taken together, the purpose of the present study was to determine the effects of an above-knee CG on passive joint position sense in the right dominant and left non-dominant knee. The second aim was to determine the magnitude of soft tissue compression produced by an above-knee CG using magnetic resonance imaging (MRI). Based on the preponderance of studies showing positive effects of CG on motor performance and proprioception, we hypothesized that 1) an above-knee CG may reduce knee joint position sense errors, 2) it may affect the dominant- and non-dominant leg’s position sense differently and 3) the pressure produced by the garment reduces the cross-sectional area (CSA) of the thigh.

## Materials and methods

### Participants

Sample size calculations (G*Power 3.1.7 [22]) for passive position error measured in the experimental (EXP) and control (CON) conditions were based on a previous study [23] which determined the effects of bracing and positioning on passive joint position sense in healthy adults’ shoulder joint. Power analysis for repeated measures analysis of variance (rANOVA) indicated a total sample size of 12, assuming type I error of 0.05 and power of 0.80.

Based on the power analysis, 24 strongly right-side dominant healthy adults were enrolled in the study (age = 27.46 ± 4.65 years, range 22–34 years; height = 1.71 ± 0.09 m; mass = 68.25 ± 12.04 kg; 18 men). Participants performed a passive target-matching task with (EXP) and without (CON) wearing an above knee CG. Participants wore the best fitting CG of the three available sizes (D&M Co., Tokyo, Japan). Side dominance was determined based on hand and leg dominance. Handedness was determined using the Edinburgh Handedness Inventory [24], a scale that is used to measure the degree of hand laterality in daily activities such as writing, drawing, throwing, using scissors, brushing teeth, opening a box, striking a match and using a pair of scissors knife, spoon, and a broom. Leg dominance was determined by one- or two-foot item skill tests such as kicking a ball or stepping up on a chair [25]. Laterality index for both handedness and footedness were calculated by summing the number of tasks performed with the right limb and the number of tasks performed with the left limb (L) as follows: (R—L)/(R + L). Laterality index was 0.96 ± 0.13 for handedness and 0.99 ± 0.02 for footedness, showing that participants were strongly right-side dominant. None of the participants had a history of neurological or orthopaedic disorders. After giving both verbal and written explanation of the experimental protocol, participants signed the informed consent document. The study was conducted according to the declaration of Helsinki and the Tohoku University Medical Ethical Committee approved the experimental protocol.

### Experimental procedures

#### Position sense measurement

Selection of the leg first used (right dominant, left non-dominant), and application of the CG (EXP, CON) were randomized. Position sense was measured on an isokinetic dynamometer (HUMAC NORM, Computer Sports Medicine Inc., Stoughton, MA). Participants wore a blindfold to eliminate vision and the white noise in the headphones eliminated auditory cues. Participants sat on the dynamometer seat in an upright position. One leg hanged freely over the edge of the dynamometer seat and the other leg was attached to the dynamometer’s lever arm.

We measured limb proprioception by a passive limb positioning protocol [26]. Participants performed a test trial to become familiar with the task. In a random order, the dynamometer moved the leg passively from the start position of 90° knee flexion to three targets, 30°, 45° and 60° of knee flexion. Participants were asked to focus on the position of the leg. The dynamometer was programmed to move the participant’s leg attached to the lever arm passively at 4°/s toward the target angle, which was then held for 5 s before the dynamometer’s lever arm with the subject’s leg attached to it, returned to the initial starting position. After 5 s, the knee joint was passively extended again at 4°/s and participants were instructed to press the stop button at the target previously practiced. Participants received no feedback about their performance through the measurement. To maintain attentional alert, after every 5 trials participants counted backwards by seven, starting from a two-digit number selected at random by the investigator.

Each target angle was repeated five times that were then averaged to calculate a mean absolute error for each target for each participant and leg. Therefore, there were 24 data points for each condition (EXP (S1 Data), CON (S2 Data)), leg (right dominant, left non-dominant), and target (30°, 45°, 60°).

#### MRI measurement

On the day after the proprioception measurement, 18 of the 24 participants were willing to undergo an MRI measurement to determine the effects of the CG on thigh CSA. The measurement was done in the standing position (G-Scan Brio, ESAOTE, Genova, Italy) by rotating the participant by ~87° without creating the feeling of instability. 3D SHARC images of 4 mm thickness were acquired under repetition time (TR) of 28.0 ms and echo time (TE) of 14.0 ms, with a pixel size of ~0.35×0.35 mm^2^, using a dedicated thigh surface coil. First, participants lay in scanner and were moved from a supine to a standing position. The acquisition time was about 20 ± 5 min, including preparation, positioning and scanning with and without wearing the CG only on the right dominant leg.

Thigh CSA was measured at ~15cm above the upper edge of the patella guided by the contour of the rectus femoris muscle. The images were digitized to determine CSA by the ImageJ software [27] as described previously [28].

### Statistical analyses

We report the data as mean ± SD. All data were checked for normal distribution using the Shapiro–Wilk test. In case of non-normality, variables were log transformed. The analyses were done on the transformed data using SPSS Statistics Package (version 22.0, SPSS Inc., Chicago, IL) but the non-transformed data are reported. The main analysis was a repeated measures analysis of variance (rANOVA) with three repetition factors of condition (EXP; CON), leg (right dominant; left non-dominant), and target angles (30°, 45°, 60°). When significant differences were detected, the multiple comparison test (Bonferroni correction) was performed. The effects of CG on thigh CSA of the thigh was examined with a paired samples t-test. In order to determine if position sense errors were associated with the magnitude of compression produced by the CG, Pearson’s correlation was computed. Cohen’s effect size, d, was also computed as appropriate. Additionally, effect sizes of repetition factors were expressed using partial eta squared (η_p_^2^) [29]. Statistical significance was set at p < 0.05. Results were interpreted by 95% confidence intervals.

## Results

**Fig 1:**
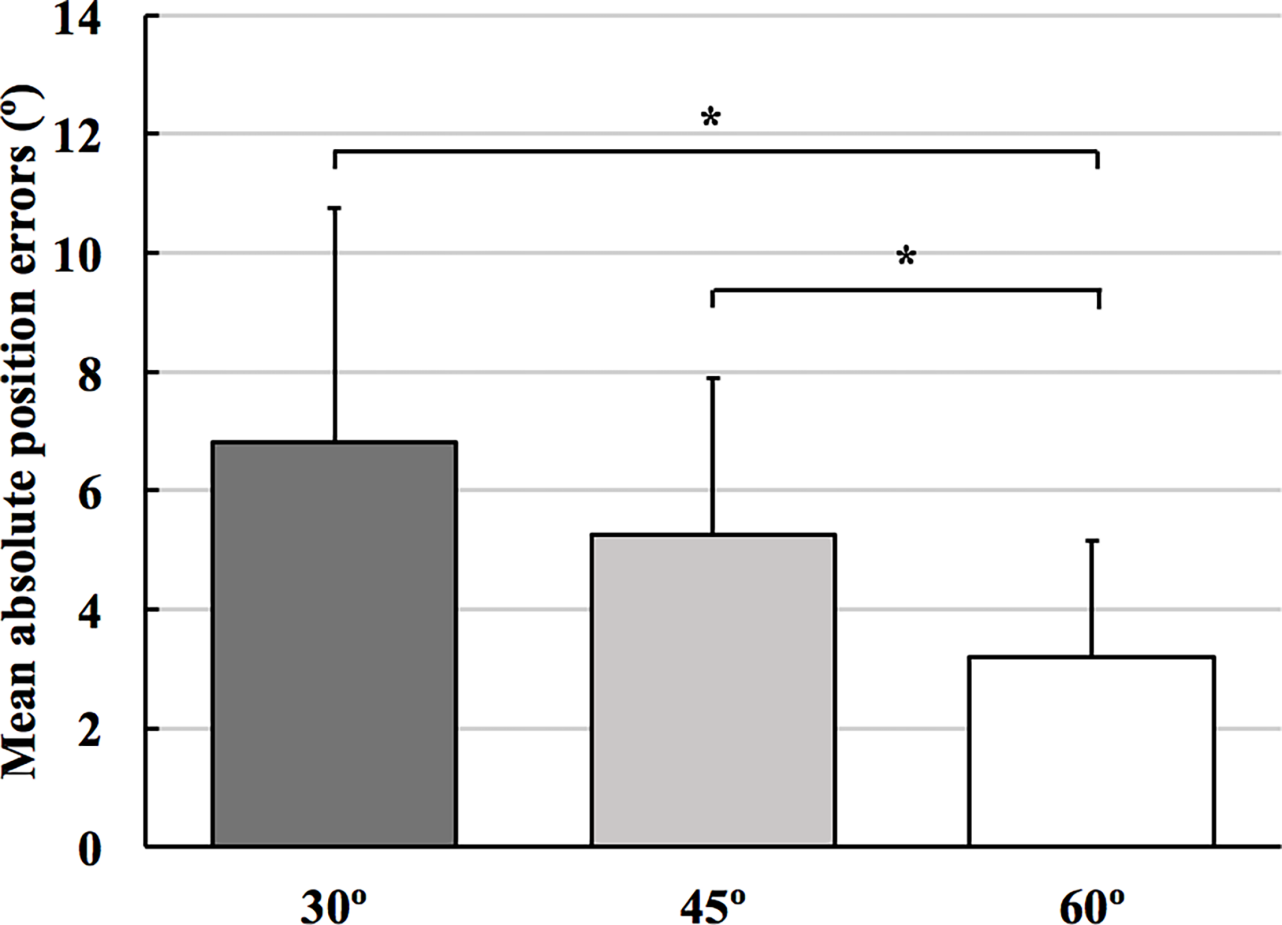
Differences in mean absolute knee joint position error at three target angles. Participants performed a passive knee target matching task with the knee joint more accurately at 60° compared to 30° and 45°. * p < 0.001.

**Fig 2:**
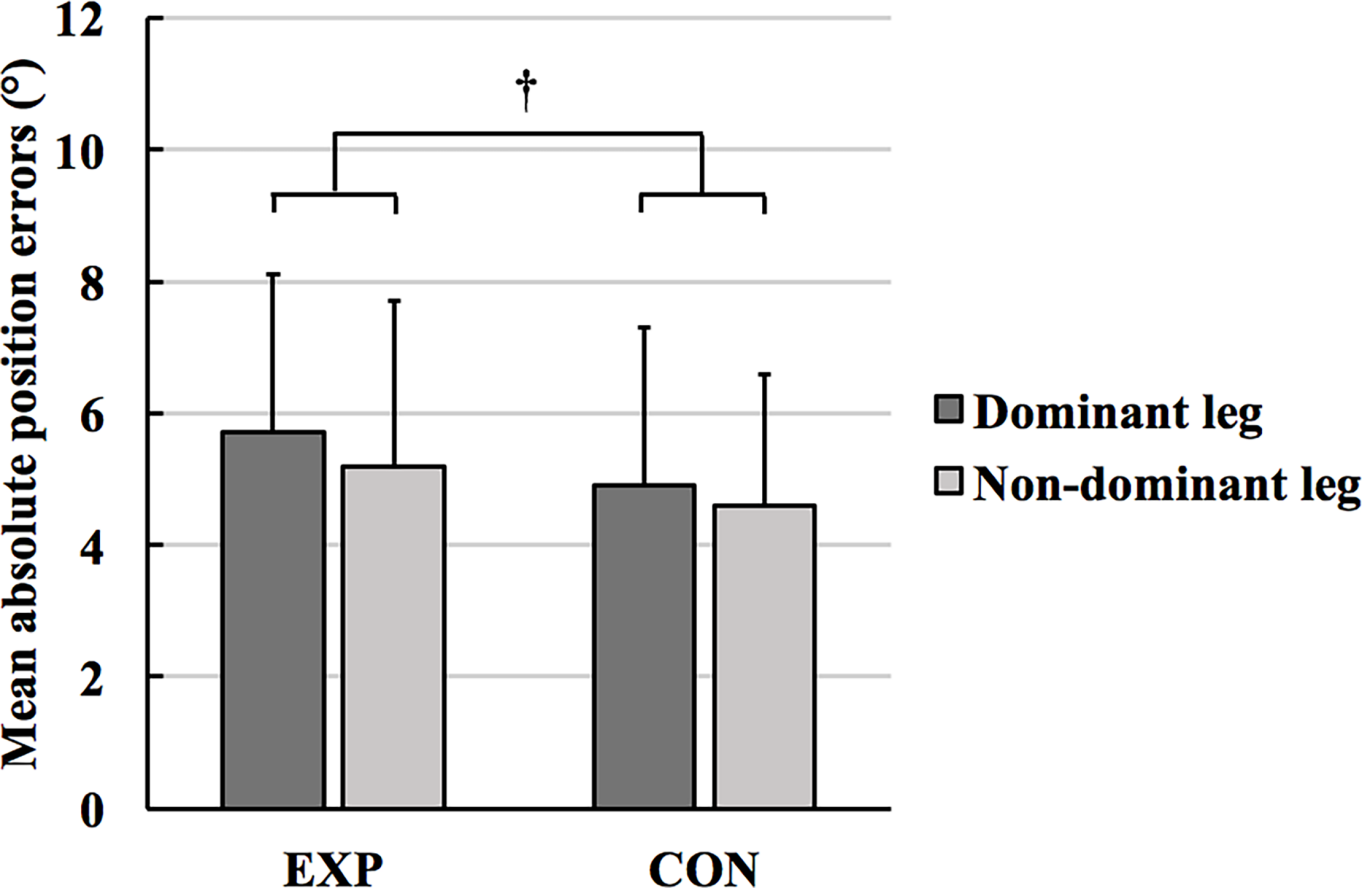
The effects of an above-knee compression garment (CG) on mean absolute position errors at the knee joint. Participants performed a position-matching task more accurately in the Control (CON) condition compared with the Experimental (EXP) condition, resulting in a significant effect of above-knee CG. † condition main effect (p = 0.014).

Table 1 shows the descriptive data for proprioceptive target-matching. rANOVA showed a main effect of target angles (F_2, 22_ = 26.569; p < 0.001; η_p_^2^ = 0.707) and condition (F_1, 23_ = 7.151; p = 0.014; η_p_^2^ = 0.237). The main effect of leg (F_1, 23_ = 0.954; p = 0.339; η_p_^2^ = 0.040) and the interaction effects of target angles × leg (F_2, 22_ = 0.083; p = 0.921; η_p_^2^ = 0.007), target angles × condition (F_2, 22_ = 0.876; p = 0.430; η_p_^2^ = 0.074), condition × leg (F_1, 23_ = 0.429; p = 0.519; η_p_^2^ = 0.018), and target angles × condition × leg (F_2, 22_ = 0.687; p = 0.513; η_p_^2^ = 0.059) were not significant. A post-hoc analysis using the Bonferroni correction revealed that accuracy of passive target matching was greater at 60° compared with 30° and 45° (p < 0.001; Fig 1). Furthermore, position errors were less in CON condition compared with EXP condition (p = 0.014, Fig 2).

**Table 1:** Mean absolute position errors obtained from a proprioceptive target matching task in the right dominant and left non-dominant legs in both conditions.

		EXP	CON
		Mean (± SD)	Mean (± SD)
Overall †		5.4 (0.9)	4.7 (1.0)
	30°	7.1 (4.0)	6.7 (4.6)
Dominant leg	45°	6.1 (2.8)	5.0 (2.5)
	60°	4.0 (2.2)	2.9 (1.8)
	30°	7.1 (4.0)	6.4 (3.1)
Non-dominant leg	45°	5.5 (2.6)	4.5 (2.6)
	60°	2.9 (1.9)	3.0 (1.8)

Values are absolute position errors (degrees). EXP: with above-knee compression garment; CON: without above-knee compression garment.

† significant condition main effect (p < 0.05).

The MRI data revealed that the garment reduced CSA by 3.2cm^2^ or 2% (CON: 187.5 ± 14.4cm^2^, EXP: 184.3 ± 13.9cm^2^, p = 0.010, Cohen’s d = 0.68). The magnitude of compression produced by the CG did not correlate with the position sense errors (p > 0.05).

## Discussion

We determined the effects of an above-knee CG on passive joint position sense in healthy adults’ knee joint and measured the magnitude of soft tissue compression produced by the garment using MRI. We found that the CG did not improve passive position sense in a target-matching task and that the CG compressed the thigh significantly but minimally by 3.2cm^2^ or 2%. Contrary to expectations, position error was less without than with the garment in the right dominant leg. These data do not support the idea that CG improves healthy adults’ joint position sense but support the notion that the type of CG we used can compress soft tissue of the thigh.

While no previous studies investigated the effect of above-knee CGs on passive joint position sense, many previous studies examined the effects of CGs on physical performance and proprioceptive position-matching errors during the task. Using a knee CG during exercise can presumably reduce microtrauma and muscular damage [30] and improve comfort [31]. In addition to knee CGs, which cover the knee joint, athletes started to use below-knee and above-knee CGs with the expectation of improving proprioception without affecting range of motion. An optimal positioning of a below-knee CG may increase Golgi tendon organ activation and feedback from proprioceptors to muscle [5, 6, 14]. Indeed, wearing a below-knee CG improved position sense in an active joint repositioning task [5]. Wearing an above-knee CG also decreased muscle oscillation in the sagittal plane during a countermovement jump test (CMJ) [32] and increased mean power output during 10 repeated vertical jumps performed by volleyball players [33]. Nevertheless, wearing an above-knee, whole leg, or a below-knee CG did not improve maximal muscular strength, jump performance, subjective feelings, and thigh/calf circumferences [34]. Combined with data from the present study (Table 1), CGs seem to affect minimally gross motor performance and as examined here, single joint proprioception.

Inconsistencies between studies make it difficult to determine if CGs could improve physical performance [17, 18] and proprioceptive acuity [5, 14, 16]. Experimental set up, participants’ training status, exercise type, garment design (e.g., knee or thigh-high stockings, waist-down tights, arm sleeves, whole body garments), the duration of exposure to CG, timing of wear (during and/or after exercise), and inflation pressure are factors contributing to the inconsistencies [35]. A limitation of the present study is that we applied only an above-knee version of CG, however, MRI data showed that participants CSA was significantly reduced when wearing above-knee CG suggesting that the pressure level by the above-knee CG was sufficient enough to produce significant changes in thigh CSA. Nevertheless, a previous review found no relationship between the effects of CGs worn during or after exercise and the magnitude of inflation pressures in the garment [7].

The current study is the first to report on the effects of above-knee CG on passive joint position sense errors. Just like a recent study [34] that investigated if wearing a lower-body CG with different body coverage areas (above-knee, whole leg, below-knee) would influence exercise performance and muscle damage, future studies need to identify if these conditions affected active and/or passive knee joint position sense. As the results from different studies are inconsistent, there is a need to probe the physiological mechanisms underlying the effect of compression on proprioceptive acuity both in healthy adults and patients with neuromuscular diseases. Although applying compression/ischemia resulted in a less accurate joint position sense by impairing afferents [10], many other studies reported that CGs could improve physical performance [36, 37] or proprioception [5, 6, 14]. Perhaps much of the favourable motor outcomes is nothing more than a placebo effect [38].

In the present study, CG failed to improve passive joint position sense of the knee joint. While a previous review suggested no relationship between the magnitude of compression by CGs and motor performance [7], we interpret the 2% compression of the thigh as insufficient to afford meaningful physiological changes regardless of a compression effect per se. Even cutaneous effects seem trivial, suggesting that CGs, as employed here, influence Ia afferent functions ineffectively when the joints are moved passively. Indeed, sensory input may increase fusimotor drive and muscle receptor activation, during active repositioning trials [39]. Such trials may also be more appropriate for functional assessment of afferent pathways due to a general attenuation and selective gating of kinesthetic awareness during active voluntary movements [40]. Muscle spindle activation appears to be higher during conscious perception of active rather than passive limb movements by detecting changes in muscle length during voluntary contractions [3]. While there were previously no data on the effects of CGs on passive proprioception and we wished to address this gap in the literature, it seems that active vs. passive repositioning measurement paradigms are more suitable to assess CGs effects on proprioception.

Target matching was more accurate at 60° compared to 30° and 45° of knee flexion. As in previous studies [14, 40], we randomized the target positions. However, it is still possible that the short path and time from the starting position of 90° to 60° required participants to explore the target in a narrower range, reducing the probability for error. In this more flexed knee position compared with 30° and 45°, the quadriceps is also more stretched, resulting in greater background Ia discharge and feedback, reducing error. A limitation of the study is that although we assessed the CSA of the leg area after the tests with the leg extended, we did not measure the pressure during the movement and in different regions under the CG.

## Conclusions

Although an above-knee CG significantly compressed the thigh by 2%, the garment did not improve proprioception in a passive knee joint position sense test. Just the opposite, in the right-dominant leg the error was actually less when it was passively moved without the garment. We measured strongly right-side dominant participants. We encourage researchers to recruit subjects with ambidexterity or “crossed laterality” (subjects with right hand-left leg or left hand-right leg dominance) to reliably determine the relationships between limb laterality and joint proprioception. Future studies should also measure the pressure and its distribution in CGs during the experimental task.

## Supporting information

S1 Data Supporting data for the experimental condition in both the right dominant and the left non-dominant legs. (XLSX)

S2 Data Supporting data for the control condition in both the right dominant and the left non-dominant legs. (XLSX)

## Abbreviations

CG: compression garment
CMJ: countermovement jump
CON: control condition, when performing position-matching task without CG
CSA: cross-sectional area
EXP: experimental condition, when placing above-knee CG during the measurement
MVC: maximal voluntary isometric contraction
rANOVA: repeated measures analysis of variance
SD: standard deviation
